# Leadership in HRH: remembering the future?

**DOI:** 10.1186/s12960-022-00738-9

**Published:** 2022-05-12

**Authors:** Inês Fronteira, James Buchan, Mario Roberto Dal Poz, Paulo Ferrinho

**Affiliations:** 1 Global Health and Tropical Medicine, Instituto de Higiene E Medicina Tropical, Universidade Nova de Lisboa, Lisboa, Portugal; 2 University of Technology, Sydney, Australia; 3 Instituto de Medicina Social, Universidade Do Estado Do Rio de Janeiro, Rio de Janeiro, Brasil

The journal has just published a paper by Ferrinho et al. [1] where the authors, based on the example of Public Health Emergencies (PHE), elaborate on the role of human resources for health (HRH) leadership not only to deal effectively with PHE but as fundamental to achieve Universal Health Coverage (UHC) by 2030.

The central issue of HRH and of effective leadership of the health workforce is not new [2]. Though it has remained a relevant topic with recurrent references in WHO´s Global strategy on human resources for health: Workforce 2030 [3], HRH leadership for the coming decades requires rethinking, to bridge the gap between what has been achieved so far and what still needs to be done to strengthen health systems and improve global health.

Two decades ago, in 2002, a group of around 100 HRH leaders called for attention to the centrality of HRH issues to respond to the Global Health Crisis and to strengthen health systems worldwide. This group became known as the Joint Learning Initiative (JLI) [4] and some of the authors of this editorial and of the paper by Ferrinho et al. [1] were active discussants in the JLI working groups and authors of several of the background papers. The JLI brought HRH to the spotlight, warned about chronic shortages, mal-distribution and a series of other persistent, regional and global, workforce problems that could seriously affect global health and the delivery of health services. It was stressed out that although health care workers were locally trained and placed, the HRH labour market was one of the most globalized and complex labour markets. In this market, health workers who face harsh working conditions—including low wages, high health-related risks (such as workplace violence, HIV/Aids or, more recently Ebola and Covid-19), high work demand and limited autonomy and recognition—and often move to where they can find better working and living conditions or abandon the health services or even the profession. This attrition is an expensive waste of highly trained professionals, aggravates shortages and results in losses of capacity and quality in delivery of health services. Then as now, the scenario was particularly appalling in low-income countries, where health systems were unable to meet the goals of Health for All defined at Alma-Ata and, more recently, the Millennium Development Goals (MDG).

Today, we know that HRH problems are not confined by geography, income or level of development and, to different degrees, are felt both in the Global South as well as in countries from the Global North. In the beginning of the twenty-first century, country leadership exerted by respected, knowledgeable leaders was considered necessary to lever strategic actions straddling from the local to the global levels of governance.

Almost as a natural evolution of the JLI, the 2006 World Health Report [5] recognized that adequate HRH were needed to accelerate progress and sustain achievements in the context of the MDGs, and called for a decade of action on HRH: the launch of the Global Health Workforce Alliance (GHWA), the convening of three global forums on HRH (GFHRH), and the introduction in 2010 by the World Health Assembly of the WHO Global Code of Practice on International Recruitment of Health Personnel are some of achievements of this period. GHWA, a “partnership of national governments, civil society, international agencies, finance institutions, researchers, educators and professional associations dedicated to identifying, implementing and advocating for solutions” to address the HRH crisis was mandated to keep HRH at the centre of the political and policy agendas [6] and assumed the responsibility for the first two Global Health *Fora*.

The Agenda for Global Action from the Kampala Declaration (AGA/KD) from the first GFHRH identified key actions (e.g., coherent national and global leadership, informed response based on evidence and joint learning, scaling up education and training, retain and equitably distribute HRH, migration and investment) required to improve HRH in the coming decade at both local, national, regional and international levels [7]. Again, coherent, “extraordinary”, leadership, focused on finding solutions was recognized as one of the strategies for action on HRH and this “new kind” of leadership should be exercised by government, professional associations, multilateral and international agencies and stakeholders in general.

The GFHRH (2011) in Bangkok reviewed progress on AGA/KD and renewed the commitment to strengthening the global health workforce, restating that a robust health workforce is a core element of health systems in all countries, and critical to achieving the MDG and UHC, with the vision that “All people, everywhere, shall have access to a skilled, motivated and supported health worker within a robust health system”. The Bangkok Outcome Statement emphasized that “Leadership by all state and non-state actors at global, regional, national and local levels is required to focus action on the health workforce”.

The 3rd GFHRH, held in 2013 produced The Recife Political Declaration on Human Resources for Health where health is recognized as a fundamental right of every human being, poor health and ill-health states are acknowledge as the “root causes of vulnerability and poverty”, where renewed vows were made towards UHC and HRH pointed out as having “an indispensable role in attaining health goals such as the Millennium Development Goals” [8]. With commitments from signing countries made at both country and international level, leadership although noticeable between the lines, seems to have faded.

2016 saw the launching of The Global Strategy on Human Resources for Health: Workforce 2030 and the UN High-Level Commission on Health Employment and Economic Growth’s report on Working for Health and Growth. Both are vital road maps to ensure an adequate leadership to achieve a fit-for-purpose and sustainable workforce to address current and future health needs. The establishment in May 2016 of the Global Health Workforce Network, hosted within WHO, was a means of leveraging multi-sectoral and multi-stakeholder engagement to advance coordination and alignment in support of the Global Strategy and High-level Commission recommendations and immediate actions, especially through facilitating information exchange and dialogue. One of its strategic objectives is “to build the capacity of institutions at sub-national, national and international levels for effective leadership and governance of actions on HRH”.

The 4th GFHRH provided a key opportunity to discuss and debate approaches towards advancing the implementation of the Global Strategy and the Commission’s recommendations, and reflect a collective commitment to developing and making available the workforce required to deliver the recently adopted Sustainable Development Goals. The Dublin forum further “urges WHO to strengthen the governance and leadership of human resources for health through the development of normative guidance, the provision of technical cooperation, and the fostering of effective trans-national coordination, alignment and accountability in order to accelerate the intersectoral implementation of the Global Strategy towards achieving its overall goal”.

The recent PHE once more brought the spotlight on HRH and emphasized the need to reflect on and promote effective HRH leaderships. Societies develop through evolution and through disruption, being the last less common and usually more visible in its effects. The Covid-19 PHE (and those to come) is a disruption in our societies and our daily lives [9] and has resulted in visible changes in health systems (e.g., change in provision of care, new skill-mix). The recent war between Ukraine and the Russian Federation is also a disruption that sooner than later will be felt in European societies and their social systems, including the health system. As such, the health system should be prepared to accommodate disruptions to innovate. To do that, leadership, as a common denominator to advance towards effective HRH policies and strengthening of health systems, should assume a pivotal role and countries should work on the development of effective, responsible and extraordinary leadership, capable of advance global health.

The agenda of a resilient and fit-for-purpose workforce cannot advance without proper attention to leadership. How can HRH be managed no matter the level (i.e. political, strategical, operational) without strong leadership? And how can all of us contribute not only to the discussion around leadership but to effectively develop strong HRH leaderships? The paper by Ferrinho et al. provides some clues but more is needed, as the predominant discussion around leadership, especially in HRH, has been essentially focused on the “what” (i.e. what is, what should be) rather than on the how. So, the question is: how to create “extraordinary” leaders in HRH without exerting too much pressure on the leaders themselves? How can countries support effective leadership without focusing on the capability of a single leader that “bears the burden of transforming an entire system”?

Leadership must facilitate the adaptation to complex environments and contexts, identifying what is wrong and to forge timeous solutions [10]. To do that leaders must have access to tools as proposed in 2002 by Van Lerberghe et al. that called for the need to develop instruments for expedient HRH impact assessments [11].

We would like to advance a little bit further and propose that not only changes, evolutions and disruptions in societies and health systems in particular should account for the impact in HRH, but also how it can affect and assist effective leadership. As such the future of leadership in HRH is in what we have learned in the past, with evolution and disruption, and how we can respond to what we experience in the present to build the future.

## Data Availability

Not applicable.
